# Methodological issues for determining intervals of subsequent cancer screening

**DOI:** 10.4178/epih/e2014010

**Published:** 2014-07-30

**Authors:** Jong-Myon Bae

**Affiliations:** Department of Preventive Medicine, Jeju National University School of Medicine, Jeju, Korea

**Keywords:** Cancer screening, Guideline, Monte Carlo method, Sensitivity, Incidence

## Abstract

The gap between nationwide recommendations of cancer screening and the related evidences obtained from Korean adults should be filled. Estimation of the mean sojourn time (MST) in a specific cancer is important to determine the intervals of subsequent screening. This author arranged the methods for calculating MST into 5 categories based on the parameters used. Under the legal barrier for protection of individual privacy and confidentiality in a Korean academic situation, the methods involving the use of transition rates or prevalence/incidence ratio would be applicable among these methods.

## INTRODUCTION

The leading cause of deaths in Korean as of 2012 is malignant neoplasm (hereafter cancer). Cancer is the primary culprit of the national disease burden as it results in more deaths than that caused by cardiac disorders and cerebrovascular diseases (the second and third causes of death, respectively) combined [1]. Various policies for cancer control are being considered around the world, and South Korea is also running a national cancer screening campaign focused on the major 5 cancers along with primary prevention, such as recommending healthy lifestyles [2]. It is important to determine screening intervals for normal-risk healthy people who have not been diagnosed with cancer in order to detect cancer early, because early cancer detection decreases death rates by the cancer [3]. In addition, screening intervals affect the cost-effectiveness of the screening project as well as screening compliance [4].

Understanding the natural history of a specific cancer is essential to determine a valid screening interval for normal-risk groups [5]. Therefore, randomized clinical trials or prospective follow-up studies to track cancer progression will provide valid grounds for establishing screening intervals. It is no exaggeration to say that the intervals for the major 5 cancers (gastric, colorectal, breast, cervical, and liver cancer) suggested by the screening program for the national cancer screening project, are not based on Korean studies. As of July 2014, Korean studies that provide grounds for 2-year screening intervals for gastric cancer are rare [6,7], and the 2-year interval suggested for breast cancer is based on the randomized comparative clinical studies on western Caucasian women, who have about 3 times the incidence rate compared with Korean women [8].

In order to achieve the goal of the national early cancer detection project, which aims to decrease deaths by cancer, studies for Koreans are vital. Thus, some methodological reviews to deduce screening intervals needs to be established. The purpose of this review is to establish and suggest some research methodologies that fit the situation of South Korea to generate a basis for determining cancer screening intervals.

## CONCEPTS RELATED TO CANCER SCREENING: MEAN SOJOURN TIME AND INTERVAL CANCER

### Mean sojourn time

**Figure 1.**
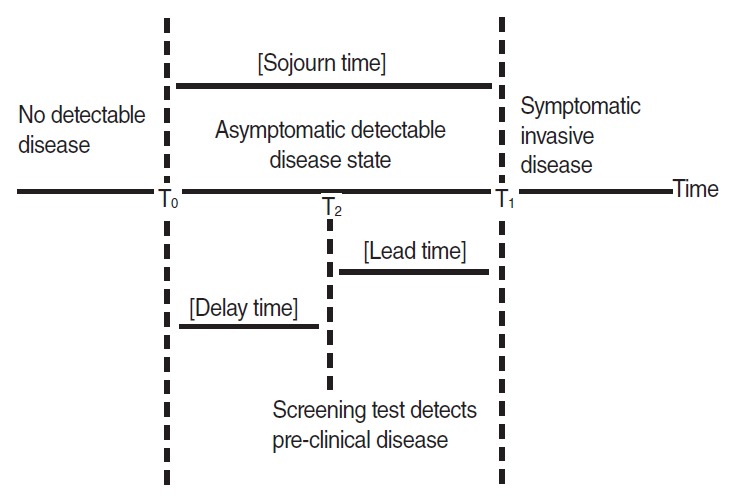
Schema for the progression of a chronic disease, with the intervention of a screening test for early detection. From Zelen M, et al. Biometrika 1969;56:601-614 [10].

Cancer, which is generated by somatic mutation during cell division [9], can be defined by 2 time points (Figure 1) in relation to process of clinical diagnosis [10,11]. The first is the point potentially detectable for cancer via screening test, T_0_, during the cell differentiation process after cancer cells have generated. Periods prior to T_0_ are those in which there is no detectable disease. The second is the point detectable for cancer via clinical symptom or signs, T_1_, when they appear owing to cancer cell division. Thus [T_0_-T_1_] is the detectable pre-clinical phase (DPCP) in which cancer can be detected early through tests, although there are no clinical signs; this period in terms of time is called as the sojourn time (ST) of a certain cancer. For instance, if a member of the normal-risk group is diagnosed with gastric cancer after screening with a gastro-fiberscope, this gastric cancer was discovered in the DPCP, prior to appearance of any clinical signs. In addition, as the subject acquired knowledge as early as the time difference between the date of screening (T_2_) and T_1_, this is defined as the lead time. Therefore, the maximal lead time for members of the normal-risk group is considered the ST by minimizing delay time.

To estimate ST of a certain cancer, the previously mentioned T_0_ and T_1_ need to be measurable; this, however, is a theoretical concept that is actually impossible to measure. As ST cannot be measured in reality, we instead measure the average ST i.e., the mean sojourn time (MST) of each subject through observations of whether cancer has occurred [12]. This MST value is the direct statistical parameter that determines screening intervals for the general public [13-15].

### Interval cancer

Interval cancer occurs when a normal-risk group, previously negative for cancer, is diagnosed with cancer within the screening interval suggested by the screening program [16]. For instance, this pertains to subjects who, with a 2-year screening interval, were determined to be cancer-negative at the time of their last gastroscopy screening, but were then diagnosed with gastric cancer in less than 2 years from the last screening. If the subject was diagnosed with cancer after more than 2 years, it is not considered interval cancer.

As such, while the definition of interval cancer varies according to screening intervals, it is also affected by the accuracy of the screening modalities [17]. Therefore, studies on interval cancer have become an important index in determining the quality of the cancer screening program and an important factor in determining appropriate screening intervals and modalities [3,18].

## ANALYTIC METHODS FOR SCREENING INTERVALS

Methods of determining screening intervals can be organized into 5 categories according to the basis of calculation.

### Based on the doubling time of the relevant cancer cell

This is based on the argument that, after calculating the minimal size detectable by current screening methods, size is determined by the doubling time of the relevant cancer cell. In other words, the screening interval is determined by the differentiation speed of the tumor. For example, the doubling time of liver cancer is on average approximately 120 days. Considering small liver cancers take approximately 5 months to grow from 1 cm to 3 cm [19], along with economic feasibility and effectiveness of the screening, the current national cancer program suggests a screening interval of 6 months for high-risk group [20,21].

In addition, the epidemiological characteristics of certain pathological types of cancer are utilized in determining screening interval. For example, the Lauren class of intestinal-type gastric cancer, which is greatly influenced by *Helicobacter pylori* infection and mucosal change, is known to have a slow progression speed; therefore, the need for conducting screening less than 2 years for subjects with atopic gastritis and intestinal metaplasia has been questioned [22].

### Based on differences between cancer stage and death rates per screening interval

The purpose of the cancer screening programs is to decrease death rates by certain cancers and increase survival rates [23]. The ideal study would be to randomly assign screening intervals, run screening tests, and then determine screening intervals by observing differences in cancer stage at the time of screening or by comparing tracked and confirmed death rates. The problem is that comparative studies through random assignment become difficult to conduct in terms of medical ethics if cancer screening has been provided as a guideline and disseminated to the public. One way to overcome this is to retrospectively confirm screening intervals prior to cancer diagnosis and compare death rates or stages.

For example, Morii et al. [24] argued that a 2-year screening interval is in fact appropriate; gastric cancer patients who underwent gastroscopy within 2 years were all diagnosed with early gastric cancer, with a 5-year survival rate of 96.5%, while those whose screening intervals exceeded 2 years had a 5-year survival rate of 71.0%, showing a significant difference. Conversely, Shiratori et al. [25] suggested a screening interval of 1.5 years; among patients diagnosed with cancer, subjects with a screening history within 1.5 years had a significantly higher diagnosis rate of early gastric cancer than advanced gastric cancer. In a Korean study, Nam et al. [26] argued for a 2-year screening interval based on the fact that while 96% (=25/26) of patients were diagnosed with early gastric cancer when taking a reexamination after receiving gastric cancer screening within 2 years, only 71% (=34/48) were diagnosed with early gastric cancer if they did not receive a gastric cancer screening within 2 years (p=0.01).

### Based on interval cancer occurrence

This method determines screening intervals by examining the interval that minimizes interval cancer occurrence. In a study of Koreans, Nam et al. [6] found that the occurrence of interval cancer significantly increased when screening in 4-5-year intervals compared to 1-year intervals, while 2-3-year intervals showed little difference from 1-year intervals; this suggested a screening interval of 3 years or less. In a study on colorectal cancer, Brenner et al. [27] conducted a screening colonoscopy cohort study in Germany. Among the 533 participants whose first colonoscopy was negative, no one was later diagnosed with colorectal cancer during the mean follow-up period of 11.9 years, and the prevalence rate of advanced adenomas was significantly lower in these patients than in those who had not received a colonoscopy for more than 10 years. Therefore, the study argues that when conducting a screening colonoscopy on a normal-risk group over 50 years in age, if no colorectal adenoma or cancer has been discovered, a screening interval of at least 5 years is appropriate.

### Based on test sensitivity and statistical models for incidence rate

As aforementioned, the methods of determining interval by comparing cancer death rates, stage at time of diagnosis, and interval cancer incidence rate require the researcher to artificially establish a screening interval in advance and reflect it in the research plan. Such limitation can lead to somewhat confusing conclusions, such as screening intervals of 2 years for Nam et al. [26] and less than 3 years for Nam et al. [6] in studies on gastric cancer screening intervals in Korean adults. This can be overcome by developing and applying various statistical model methods for estimating MST directly.

Attempts to directly estimate MST started after Zelen and Feinleib [10] suggested the DPCP concept along with the MST formula in 1969. The article by Shen and Zelen [28] reviewed various models using the statistical method called Markov Chain Monte Carlos (MCMC). In South Korea, using the threshold model suggested by Lee and Zelen [29] in 1998, Jung et al. [30] and Lee et al. [31] attempted to calculate MST in breast cancer. In reality, however, the attempts were limited because the method was only applicable for calculating MST only if the cancer incidence rates in subjects who have not been screened and the sensitivity of the screening are known. The two domestic studies were both limited because they used the incidence rate and sensitivity of Caucasians rather than Koreans.

### Based on the interaction formula between prevalence and incidence rates

Another research task is to measure the sensitivity of the early diagnostic screening method in cancer screening. This is because determining sensitivity, which pertains to the ability to detect subjects with illness in screening [32], is only possible if one can acquire information on either cancer occurrence or death through complete follow-up studies. In other words, determining the sensitivity of cancer screening methods can actually be impossible in Korean academic society now. Two convenient methods of calculating MST when sensitivity is not given have been suggested below.

#### Utilizing the transition rate

In 2011, Brenner et al. [14] suggested a transition rate formula by using the interaction formula between prevalence rate and incidence rate when it satisfies the suggested premises. The premises were that both screening participants and non-participants have the same prevalence rate for a certain cancer, and that the incidence rate and transition rate in the screening participants is constant during the observation period. As such, transition rates could be calculated using screening participation rates, prevalence rates, and incidence rates, even though the sensitivity is not known. The calculated transition rate pertains to the speed of transition from DPCP to the clinical diagnosed status, and as such, the reciprocal of the transition rate becomes the MST.

As cancer screening is conducted in the ordinary person prior to disease emergence, not only are most of the premises of the formula derived by Brenner et al. [14] satisfied, but also another advantage is that the required parameters can be acquired in the process of screening. The problem is that follow-up information describing whether screening participants were diagnosed with cancer is required for calculating cancer incidence rates in the transition rate formula at the time of induction. Although, considering the situation in South Korea, incidence rates can be acquired through the Central Cancer Registry database of the National Cancer Center that registers all cancer patients, the information cannot be utilized by general researchers owing to legal restrictions on personal information protection.

Considering the characteristics of the screening sites that conduct biopsies in cases of suspected cancer, which are based on observation during gastroscopy for early detection of gastric cancer, pathologic findings rather than gross findings can be a basis for acquiring cancer incidence rates. On the other hand, as in mammography for early detection of breast cancer, tests for definite diagnosis are performed at a different time and location after imaging and the medical scientist has given his interpretation; therefore, diagnosis data alone cannot be used to calculate incidence rates. Considering the execution characteristics of gastroscopy, in an attempt to calculate the gastric cancer MST in Korean men, Bae et al. [7] organized data on screenees who repeatedly underwent thorough gastroscopy screening, calculated prevalence rates using the first screening results, obtained repeated screening participation rates, and then used biopsy results to calculate the incidence rate.

#### Borrowing results of existing studies

In 2013, Draisma and von Rosmalen [33] suggested a simplified formula that states MST is the prevalence rate on the DPCP divided by incidence rate (I). Here, prevalence rate on the DPCP is calculated by dividing the positive detection rate (R) by the sensitivity (S). In other words, MST can be obtained if I, R, and S are given. Following this simplified formula, the authors borrowed incidence rates from existing studies to obtain the MST of prostate cancer.

When applying this method after establishing a cohort data that follows screening examinees, the positive detection rate at the first screening can be calculated and the incidence rate (I) can be acquired using national cancer data; then MST can be calculated by using the sensitivity suggested in existing papers. For instance, according to the study by Nam et al. [6] on screening gastroscopy, 48.9% of examinees diagnosed with gastric cancer at the National Cancer Center between 2004 and 2009 were reported to have undergone thorough gastric cancer diagnosis in the past. If these data are utilized, then 2.05 times the number of positively diagnosed examinees (=1/0.489) can be considered to have newly generated cancer. In other words, as 48.9% of the actual cancer patients were positively diagnosed via screening, multiplication with the reciprocal of 48.9%, that is 2.05 (w), is the estimated number of incidences. Bae et al. [34] applied such concepts in an attempt to calculate gastric cancer MST.

## CONCLUSIONS AND SUGGESTIONS

It is a fact that current national cancer screening guidelines lack supporting grounds. To determine screening intervals of early cancer detection, research on MST is vital. The present paper has divided MST calculation methods into 5 categories and observed the requirements for computation. In conclusion, to accurately calculate MST, incidence rate must be calculable, and sensitivity according to subject’s characteristics must be obtainable from the National Cancer Center. We hope to be able to conduct research of public value in line with the purpose of the ‘Act on Vitalizing Supply and Usage of Public Data’, enacted as of October 31, 2013.

In reality, however, with the legal barriers of personal information protection limiting opportunities to conduct public research, such as MST calculation, utilizing the 2 simplified formulas belonging to the last categories may be an option. In particular, considering the author’s experience in attempting to calculate MST for gastric cancer through gastroscopy, the author would like to suggest attempting MST calculation for colorectal cancer in Koreans by using colonoscopy, despite the difficult environment in conducting research.
