# Supplementary material for: Methodological issues for determining intervals of subsequent cancer screening
Source: Epidemiol Health. 2014 Jul 30;36:e2014010. doi: 10.4178/epih/e2014010 (PMC4153009; doi:10.4178/epih/e2014010)
Supplement: Supplementary file 1 [file epih-36-e2014010-supplemetal data.pdf]

특정 암의 검진 주기 결정을 위한 연구 방법론

Methodological issues for Determining Intervals of Subsequent Cancer screening

Abstract

The gap between nationwide recommendations of cancer screening and the related evidences obtained from Koreans should be filled. Estimating of mean sojourn time (MST) in a specific cancer is important to determine the intervals of subsequent screening. Author arranged the methods for calculating MST into 5 categories based on using parameters. Under the legal barrier for protection of individual privacy and confidentiality in Korean academic situation, the methods using transition rates or prevalence/incidence ratio would be applicable among them.

Key words: Cancer Screening, Guideline, Monte Carlo method, Sensitivity, Incidence

1. 서론

2012년도 현재 우리나라의 사망원인 1위는 악성신생물 (이하 암)로서, 2위와 3위인 심장질환과 뇌혈관질환을 합친 사망률보다도 더 높은 사망률을 보이고 있다는 점에서 국민질병부담의 주범에 해당한다[1]. 세계 각국에서는 암의 관리를 위한 다양한 정책을 강구하고 있으며, 우리나라도 건강한 생활습관 갖기 등의 일차예방과 함께, 5대 암을 중심한 국가암검진사업을 벌이고 있다[2]. 암의 조기발견을 목적으로 수행하는 암 검진사업에서 검사를 받아 음성으로 나온 일반인 (normal-risk group)이 다음 검진을 언제 해야 하는가? 라는 검진 주기 (screening interval)를 결정하는 것은 암 사망률을 줄이려는 암검진의 목적 달성에 핵심적인 사안이다[3]. 왜냐하면 검진주기의 결정은 사업의 비용-효과 (cost-effectiveness) 측면뿐만 아니라, 수검의 순응도 (compliance) 측면에서 결정적인 영향을 주기 때문이다 [4].

건강한 일반인을 대상으로 타당한 검진 주기를 결정하기 위해서는, 해당 암의 진행과정을 이해하는 것이 중요하다[5]. 따라서 환자 중심의 사례연구보다는 진행과정을 추적하는 코호트 관찰연구나 무작위비교임상시험을 수행하여야 주기 결정의 타당한 근거를 만들어 낼 수 있다. 그러나 국가암검진 5대 대상 암들 – 위암, 대장암, 유방암, 자궁경부암, 간암-에 있어 검진지침에서 제시된 주기는 한국인을 대상으로 한 연구결과에 근거하여 결정한 것은 없다고 해도 과언이 아니다. 2014년 7월 현재 한국인을 대상으로 위암의 2년 주기 결정에 대한 근거를 제공하는 연구는 희소하며[6,7], 유방암의 2년 주기 제시는 한국여성보다 3배 이상 높은 발생률을 가진 서구 백인여성에서 얻어낸 무작위비교임상시험 연구결과를 근거로 하고 있기 때문이다[8].

따라서 암사망률을 낮추려는 국가암조기검진사업이 그 목적을 달성하려면, 한국인을 대상으로 한 질 높은 연구가 활성화되어야 하지만, 동시에 검진 주기를 도출할 수 있는 연구방법론도 정립되어야 할 것이다. 이 종설의 목적은, 암검진의 주기결정의 근거 생성을 위해 한국의 실정에 맞는 연구방법론을 정립, 제시하는 것이다.

2. 암 검진에 관련한 개념 정리: 간격암, 평균체류기간

가. 평균체류기간 (Mean Sojourn Time, MST)

세포분열과정에서 'somatic mutation'에 의하여 발생되는 암은[9], 발생에서 임상적으로 진단되는

전체 과정과 관련하여 그림 1처럼 두 가지 시점을 정의할 수 있다 [10, 11]. 첫째 시점은 암세포 발생 후 분화과정에서 개발된 검사방법을 동원하여 검출이 가능한 시점 (Tumor potentially detectable by screening test, T_0)이다. 따라서 T_0 이전의 기간은 검출불가능 (no detectable disease) 구간이 된다. 둘째 시점은 암세포의 분화로 인하여 임상 증상이 나타나서 임상적으로 진단이 가능한 시점 (Tumor detectable clinically, T_1)이다. 이때 [T_0 – T_1] 구간은 임상증상이 없지만 검사를 통해 조기에 암을 발견할 수 있는 전(前)임상구간 (Detectable Pre-clinical Phase, DPCP)이며, 이 구간을 시간으로 표시한 것이 바로 특정 암의 체류기간 (Sojourn Time, ST)이 된다. 예를 들어 건강한 일반인에 있어 위내시경을 시행하여 위암을 발견했다면, 이 대상자는 임상증상이 나타나기 전인 DPCP에서 위암을 조기에 발견한 것이며, 검진일 (T_2)과 T_1 간의 시간 간격만큼 미리 알게 되었다는 점에서 선행시간 (Lead Time)라 정의한다. 따라서 선행시간의 최대값이 ST가 되는 것이다.

그런데 특정 암의 체류기간을 알아내려면 앞서 언급한 T_0 와 T_1 두 가지 기준 시점을 측정할 수 있어야 하는데, 이 둘 다 현실적으로 측정이 불가능한 이론적 개념이다. 이렇게 실제적으로 측정이 불가능한 ST에 있어서, 대상자 추적관찰을 통해 암발생 여부에 따라 각각 얻어낸 체류시간의 평균값 – 즉 평균체류기간 (Mean Sojourn Time, MST) –을 산출하게 된다 [12]. 바로 이 MST값이 일반국민을 대상으로 한 검진 주기를 결정하는 직접적인 통계치인 것이다 [13-15].

나. 간격암 (Interval Cancer)

간격암은 검진 음성으로 판정된 일반인이 검진 지침에서 권고하는 검진 주기 내에 해당 암 발생으로 진단받은 경우를 말한다 [16]. 예를 들어서 위암 검진 주기가 2년인 상황에서, 가장 마지막으로 받은 검진용 위내시경에서 음성으로 판정을 받았으나 그 시점 이후 2년이 안된 구간 중에 위암으로 진단을 받은 대상자를 말한다. 만약 2년이 넘어서서 위암 진단을 받은 대상자는 간격암이 아니다.

이렇듯 간격암은 검진주기에 따라 정의를 달리하게 되지만, 검사의 정확도에도 영향을 받는다 [17]. 따라서 간격암 연구를 통해 암 검진 프로그램의 질을 결정하는 중요한 지표가 되면서, 해당 검사의 주기와 방법을 결정하는 중요한 요소이다 [3, 18].

3. 검진 주기 분석 방법들

암검진의 주기를 결정하는 방법들은 적용하는 산출 근거에 따라 크게 5가지 범주로 정리할 수 있다.

가. 해당 암 세포의 배가 시간 (doubling time)를 근거

현재의 검진방법으로 검출이 가능한 최소 크기를 산정한 뒤, 그 크기가 해당 암세포의 분화 속도

에 의해 결정된다는 주장에 근거한 것이다. 다시 말해서 종양의 분화 속도에 따라 검진 주기를 결정한다는 뜻이다. 그 예 중의 하나로 간암의 배가 시간은 평균 약 120일이며, 크기가 작은 간암의 경우 1cm에서 3cm 까지 자라는데 빠르게 자랄 때 약 5개월이 걸린다는 사실에 따라 [19], 종양의 부피와 배가 시간, 조기검진으로서의 경제성과 효과를 고려하여, 현재 국가암지침은 고위험군에 대해 6개월 간격의 검진 주기를 권하고 있다 [20, 21].

또한, 특정 암에서 병리학적 유형의 역학적 특성에 근거하여 검진주기를 판단하는데도 활용하고 있다. 그 예로는 *H.pylori* 감염과 점막변화에 영향을 많이 받는 Lauren 분류의 장형(intestinal type) 위암인 경우 그 진행 속도가 늦다고 알려져 있어서, 위축성 위염 (atopic gastritis)과 장상피화생 점막 병변 (intestinal metaplasia)을 갖고 있는 대상에서 위암 검진을 2년 보다 더 자주 받는 것에 의문을 제기하였다 [22].

나. 검진주기별 암 병기 및 사망률 차이에 근거

암의 조기검진사업의 궁극적인 목적이 특정 암에 의한 사망률을 줄이고 생존률을 높이는 것이다 [23]. 이를 위해서는 검진주기별로 무작위 배정하여 검진을 시행한 다음, 검진 당시 병기 (stage)의 차이를 보거나, 사망통계를 활용하여 추적 확인한 사망률의 차이를 비교하여 적절한 검진주기를 결정하는 것이 이상적이다. 문제는 암 검진이 지침으로 제시되어 일반인들에게 이미 확산된 상황이라면, 무작위배정을 통한 비교연구는 의료윤리적으로 수행하기 어려운 상황에 봉착하게 된다. 이를 극복하는 방법의 하나로, 암 발생환자를 중심으로 암 진단 전 검진주기를 후향적으로 알아내어 사망률이나 병기의 차이를 비교하는 것이다.

이에 대한 예로써, Morii 등 [24]은 2년 내에 내시경 검사를 받은 적이 있는 위암 환자는 모두 조기위암이었으며 5년 생존율이 96.5%로 나타나서, 2년 이상 된 군의 71.0%에 비해 유의하게 높았다고 보고하면서 이를 근거로 검사주기가 2년이 적절하다고 주장하였다. 반면, Shiratori 등 [25]은 검진을 받았던 경험이 있었던 경우 중 1년 반 이내에 검진 병력 (screening history)이 있는 경우 진행성 (advanced) 위암에 비해 조기 (early) 위암의 진단율이 유의하게 높았기 때문에 검진주기를 1년 반으로 할 것을 제안하였다. 한국인을 대상으로 한 연구로 남수연 등 [26]은 2년 이내에 위암검진을 받은 후 재검하는 경우에는 96% (25/26)에서 조기위암이었던 반면, 2년 이내에 위암검진을 받지 않은 경우에는 71% (34/48)에서만 조기위암이었다($p=0.01$)라는 결과를 바탕으로, 2년 주기를 주장하였다.

다. 간격암 (interval cancer) 발생 여부를 근거

간격암 발생을 최소화할 수 있는 검진 간격을 알아봄으로써, 검진주기를 결정하는 것이다. 우리나라 국민을 대상으로 한 예로써, 남지형 등 [6]은 위암 환자-대조군 연구를 통해 1년 주기에 비해 4-5

년 주기로 할 경우 간격암 발생이 유의하게 증가하며, 2-3년의 주기로 할 경우는 1년 주기와 차이가 없었다고 결과에 따라 3년 이내의 검진 주기를 제안하였다. 대장암에 대한 예로써, Brenner 등 [27]은 독일의 선별대장내시경검사 (screening colonoscopy) 코호트 연구를 수행한 결과, 첫 대장내시경검사가 음성이었던 533명의 연구 참가자 중 평균 11.9년의 추적검사 기간 동안 대장암이 발생한 예는 하나도 없었으며, 진행샘종 (advanced adenomas)의 유병률도 대장내시경을 받지 않은 사람에 비해 10년 이상 유의하게 낮은 상태로 유지되고 있었다. 따라서 50세 이상의 평균위험군을 대상으로 선별대장내시경검사를 시행하였을 때 대장샘종이나 대장암이 발견되지 않은 경우에는 다음 검사 시기를 최소 5년 이상으로 하는 것이 적절하다고 주장하였다.

라. 검사 민감도와 발생률의 통계 모형에 근거

앞서 언급한 것에서 암 사망률, 진단시 병기, 간격암 발생률의 비교를 통해 주기를 결정하는 연구방법은 연구자가 검진주기를 사전에 인위적으로 정한 다음, 이를 연구계획서에 반영해야 한다. 이런 어려움에 따라, 한국 성인의 위암에 대한 검진주기 연구에서 남수연 등[26]은 2년으로, 남지형 등 [6]은 3년 이하라는 다소 혼란스런 결론을 내는 상황이 연출될 수 있다. 이를 극복하는 방법은 다양한 통계모형기법을 개발, 적용하여 MST를 직접 추정하는 것이다.

MST를 직접 추정하는 시도는 1969년 Zelen & Feinleib [10]이 DPCP 개념을 제시하면서 MST 산출식을 제시한 이후 본격화 되었다. 이후 Markov Chain Monte Carlos (MCMC)란 통계법을 활용한 다양한 모형 구축시도에 대하여는 Shen & Zelen의 논문[28]을 참고하길 바란다. 추가로 언급할 것은 1998년 Lee & Zelen [29]이 제시한 threshold 모형을 활용하여 국내에서는 유방암에 대하여 정성화 등 [30]과 이순영 등 [31]이 MST 산출을 시도했다는 점이다. 그렇지만, 해당 모형을 이용하여 MST를 산출하기 위해서는 두 가지 수치 - 검진의 민감도와 검진을 받지 않는 대상에서의 암발생률 -를 알아야만 적용이 가능하다는 현실적인 한계를 갖는다. 국내의 두 연구 모두는 한국인이 아닌 서구인의 발생률과 민감도를 차용하는 한계를 가지고 있다.

마. 유병률과 발생률의 관계식에 근거

암검진에서 조기진단용 검진법의 민감도를 측정하는 것은 또 다른 연구 과제에 해당한다. 민감도는 질병을 가진 대상을 검진에서 검출해내는 능력을 의미하는 민감도 [32]를 알아내기 위해서는, 완전한 추적조사를 통해 암발생 혹은 사망이라는 결과 정보를 확보해야만 가능하기 때문이다. 이를 달리 말하면 암검진법의 민감도를 알아내는 것이 현실적으로는 불가능한 경우가 있다.

이렇게 실제적으로 민감도를 알 수 없는 경우에 MST를 산출할 수 있는 간편 산출방식이 다음 두 가지로 제시되었다.

(1) 전이율 (Transition rate)을 활용하는 방법

2011년 Brenner 등 [14]은 제시한 전제들을 만족할 경우 유병률과 발생률 관계식을 이용하여 전이율 산출식을 제시하였다. 가정한 전제들은 검진 참여군과 검진 불참여 군 간에 특정 암 유병률이 같으며, 검진참여군에서의 발생률과 전이율이 관찰기간 내에 일정하다는 것이다. 이에 따라 민감도를 몰라도 검진참여율, 유병률, 발생률의 3가지만으로 전이율을 산출할 수 있다. 산출되는 전이율은 DPCP 에서 임상적 진단 (clinical diagnosed) 상태로 전이하는 속도를 의미하므로, 전이율의 역수가 바로 MST가 되는 것이다.

암검진은 질병발생 이전의 일반인들을 대상으로 시행하기 때문에, Brenner 등 [14]이 유도한 수식의 전제 조건들을 만족시키는 것은 대부분 수용될 뿐만 아니라, 필요한 모수들은 일반인을 대상으로 한 검진프로그램 시행과정에서 얻어낼 수 있다는 장점을 가진다. 문제는 전이율 유도식에서 요구하는 암발생률은 검진 대상자에서 암진단 여부에 대한 추적이 가능해야만 산출할 수 있다는 점이다. 우리나라 실정상 전국민 암발생자를 등록하는 한국중앙암등록본부의 등록자료를 이용하면 발생률을 구할 수 있겠으나, 개인정보보호란 법적 장벽 때문에 일반 연구자로서는 활용이 불가능하다.

그런데, 위암 조기발견을 목적으로 시행하는 위내시경은 육안으로 암이 의심이 될 경우 거의 모든 경우에서 생검 (biopsy)을 하는 검진 현장의 특성을 감안할 때, 육안소견이 아닌 생검의 병리학적 소견을 근거로 암발생률을 구할 수 있겠다. 반면에 유방 조기발견을 위한 유방촬영술은 촬영 후 영상의학자의 판독소견이 있은 다음 다른 시공간에서 확진을 위한 검사가 이루어지므로, 검진 자료만으로는 발생률을 산출할 수 없겠다. 따라서 위내시경의 실행 특성을 감안해서 배종면 등 [7]은 위내시경을 통한 위암 조기검진을 반복 시행한 검진자를 대상으로 자료를 정리한 다음, 첫 검진결과를 이용하여 유병률을 산출하고, 반복 검진의 참여율을 얻어내며, 생검 결과를 발생률 산출에 활용하여 한국 남성의 위암 MST 산출을 시도하였다.

(2) 기존의 다른 연구의 결과를 차용하는 방법

2013년 Draisma & von Rosmalen [33]은 PCDP 구간내 유병률을 발생률 (I)로 나누어 준 값이 바로 MST라는 간편식을 제안하였다. 여기서 PCDP 구간내 유병률은 첫 검진시 양성검출률 (R)을 민감도 (S)로 나누어 산출한다. 다시 말해서 I, R, S를 알면 MST를 구할 수 있다는 것이다. 이런 간편식에 따라, 저자들은 발생률을 기존의 연구에서 차용하여 전립선암의 MST를 구하였다.

만약 검진수검자를 추적조사하는 DB를 구축해서 이 방법을 적용한다면, 첫 검진시 양성 검출률을 직접 산출할 수 있기에 국가 암등록자료를 이용하여 발생률 I를 구할 수만 있다면, 기존의 논문에서 제시하는 민감도를 활용해서 MST를 산출해 볼 수 있다. 예를 들어, 검진용 위내시경에 있어 남지형 등 [6]의 연구에 따르면, 2004-2009년간 국립암센터 위암센터에서 위암 양성 진단을 받은 대상자 중

48.9%가 과거에 위암검진을 받은 것으로 발표하였다. 이 결과를 활용하면 검진과정을 통해 양성검출자 명수의 2.05배 (=1/0.489) 만큼이 새로이 발생되는 암이라고 추정할 수 있겠다. 다시 말해서 실제 암 환자 중 48.9%가 확인된 것이 검진으로 알게 된 양성검출자란 의미이므로, 48.9%의 역수인 2.05 (w)을 곱한 것이 예상되는 발생자 명수가 되는 것이다. 이런 개념을 적용하여 배종면 등 [34]은 위암의 MST 산출을 시도하였다.

4. 결어 및 제언

현재 국가암검진 지침에 있어 관련 근거가 부족한 것은 숨길 수 없는 사실이다. 특히 한국인을 대상으로 암조기검진의 시행 주기를 결정하기 위해서 MST 연구가 활성화되어야 한다. 지금껏 MST의 산출방법을 5가지로 대분하여 산출에 필요한 항목들을 살펴보았다. 결론적으로 MST를 보다 정확하게 직접 산출하려면 무엇보다도 중앙암등록본부의 등록자료를 통해 연구대상자에서의 발생률을 산출할 수 있어야 하며, 연구대상자 특성에 따른 민감도를 얻어낼 수 있어야 한다. 2013년 10월 31일부터 시행에 들어간 '공공데이터의 제공 및 이용 활성화에 관한 법률'의 취지에 따라 공익적 연구를 수행할 수 있기를 바란다.

그러나 현실적으로는 개인정보보호란 법적 장벽에 우선 막혀서 MST 산출 같은 공익적인 연구를 수행할 수 없는 현 상황에서, 마지막 범주에 속하는 두 가지 간편식을 적극 활용하는 것도 고려해 볼 수 있다. 특히 저자는 위내시경을 이용한 위암의 MST 산출을 시도한 경험에 따라, 연구 수행이 어려운 상황임에도 불구하고 한국인을 대상으로 대장내시경을 이용한 대장암의 MST 산출 등을 시도해 볼 것을 제안한다.

감사의 글

본 연구는 대한민국 보건복지부 암정복추진연구사업비 지원을 받음(No. 1320300).

References

1. Jung KW, Won YJ, Kong HJ, Oh CM, Lee DH, Lee JS. Cancer statistics in Korea: Incidence, mortality, survival, and prevalence in 2011. Cancer Res Treat 2014; 46: 109-23.
2. Suh MN, Cohi KS, Lee YY, Jung JK. Trends in cancer screening rates among Korean men and women: Results from the Korean National Cancer Screening Survey, 2004-2012. Cancer Res Treat 2013; 45: 86-94
3. Seo HG. Screening for early detection of cancers II. J Korean Med Assoc 2006; 49(6): 515-530. (Korean)
4. Yoo KB, Kwon JA, Cho E, Kang MH, Nam JM, Choi KS, et al. Is mammography for breast cancer screening cost-effective in both Western and Asian countries? Results of a systematic review. Asian Pac J Cancer Prev 2013: 14; 4141-9.
5. Spix C, Blettner M. Screening – part 19 of a series on evaluation of scientific publications. Dtsch Arztebl Int 2012; 109: 385-90.
6. Nam JH, Choi IJ, Cho SJ, Kim CG, Jun JK, Choi KS, et al. Association of the interval between endoscopies with gastric cancer stage at diagnosis in a region of high prevalence. Cancer 2012; 118: 4953-60.
7. Bae JM, Shin SY, Kim EH. Mean sojourn time of preclinical gastric cancer in Korean Men: A retrospective observational study. J Prev Med Public Health 2014; 47: 79-83.
8. Bae JM. On the benefits and harms of mammography for breast cancer screening in Korean women. Korean J Fam Pract 2014; 4: 1-6.
9. Burnet Macfarlane. Cancer – A biological Approach. I. The processes of control. Br Med J 1957; 1: 779-86.
10. Zelen M, Feinleib M. On the theory of screening for chronic diseases. Biometrika 1969; 56: 601-14.
11. Day NE, Walter SD. Simplified models of screening for chronic disease: Estimation procedures from mass screening programmes. Biometrics 1984; 40: 1-14.
12. Chen THH, Kuo HS, Yen MF, Lai MS, Tabar L, Duffy SW. Estimation of sojourn time in chronic disease screening without data on interval cases. Biometrics 2000; 56: 167-72.
13. Pashayan N, Duffy SW, Pharoah P, Greenberg D, Donovan J, Martin RM, et al. Mean sojourn time, overdiagnosis, and reduction in advanced stage prostate cancer due to screening with PSA: implications of sojourn time on screening. Br J Cancer 2009; 100: 1198-204.
14. Brenner H, Altenhofen L, Katalinic A, Lansdorp-Vogelaar I, Hoffmeister M. Sojourn time of preclinical colorectal cancer by sex and age: estimates from the German National Screening Colonoscopy Database. Am J Epidemiol 2011; 174: 1140-6.
15. Weedon-Fekjaer H, Vatten LJ, Aalen OO, Lindqvist B, Tretli S. Estimating mean sojourn time and screening test sensitivity in breast cancer mammography screening: new results. J Med Screen 2005;

12: 172-8.
16. Houssami N, Irwig L, Ciatto S. Radiological surveillance of interval breast cancers in screening programmes. Lancet Oncol 2006; 7: 259-65.
17. Rosenberg RD, Hung WC, Williamson MR, Gilliland FD, Wiest PW, Kelsey CA, et al. Effects of age, breast density, ethnicity, and estrogen replacement therapy on screeening mammograhic sensitivity and cancer stage at diagnosis: Review of 183,134 screening mammograms in Albuquerque, New Mexico. Radiology 1998; 209: 511-8.
18. Kim JY, Cho BH, Hur MH, Kang SS, Lee JH, Lee SK, et al. Interval breast cancers: Comparisons with Screen Detected Cancers. J Korean Surg Soc 2002; 63: 458-61.
19. Lencioni R. Surveillance and early diagnosis of hepatocellular carcinoma. Dig Liver Dis. 2010; 42 Suppl 4: S2223-7.
20. Lee MA. Screening and surveillance of hepatocellular carcinoma. Korean J Med 2010; 79: 224-30.
21. Colombo M. Screening and diagnosis of hepatocellular carcinoma. Liver Int 2009; 29 Suppl 1: 143-7.
22. Correa P. Human gastric carcinogenesis: A multistep and multifactorial process – First American Cancer Society Award Lecture on cancer epidemiology and prevention. Cancer Res 1992; 52: 6735-40.
23. Miettinen OS. Screening for a cancer: a sad chapter in today's epidemiology. Eur J Epidemiol 2008; 23: 647-53.
24. Morii Y, Arita T, Shimoda K, Yasuda K, Yoshida T, Kitano S. Effect of periodic endoscopy for gastric cancer on early detection and improvement of survival. Gastric Cancer 2001; 4: 132-6.
25. Shiratori Y, Nakagawa S, Kikuchi A, Ishii M, Ueno M, Miyashita T, et al. Significance of a gastric mass screening survey. Am J Gastroenterol 1985; 80: 831-4.
26. Nam SY, Choi IJ, Park KW, Kim CG, Lee JY, Kook MC, et al. Effect of repeated endoscopic screening on the incidence and treatment of gastric cancer in health screenees. Eur J Gastroenterol Hepatol 2009; 21: 855-60.
27. Brenner H, Haug U, Arndt V, Stegmaier C, Altenhofen L, Hoffmeister M. Low risk of colorectal cancer and advanced adenomas more than 10 years after negative colonoscopy. Gastroenterology 2010; 138: 870-6.
28. Shen Y, Zelen M. Parametric estimation procedures for screening programmes: Stable and nonstable disease models for multimodality case finding. Biometrika 1999; 86: 503-15.
29. Lee SJ, Zelen M. Scheduling periodic examinations for early detection of disease: application to breast cancer. J Am Statist Assoc 1998; 93: 1271-81.
30. Jeong SH, Kang DR, Hur NW, Kim JH, Lee SY, Jung SH, et al. A study on scheduling periodic examinations for the early detection of breast cancer in Korea. J Prev Med Public Health 2006; 39: 346-52.
31. Lee SY, Jeong SH, Kim JH, Jung SH, Song KB, Nam CM. Scheduling mammography screening for the

early detection of breast cancer in Korean women. J Med Screen 2007; 14: 205-9.
32. Kim SH, Wu D. Estimation of sensitivity depending on sojourn time and time spent in preclinical state. Stat Methods Med Res 2014. [PMID: 23129110]
33. Draisma G, van Rosmalen J. A note on the catch-up time method for estimating lead or sojourn time in prostate cancer screening. Stat Med 2013; 32: 3332-41.
34. Bae JM, Shin SY, Kim EH. Optimal interval for repeated gastric cancer screening in normal-risk healthy Korean adults: a retrospective cohort study. Cancer Res Treat 2014 (in press)

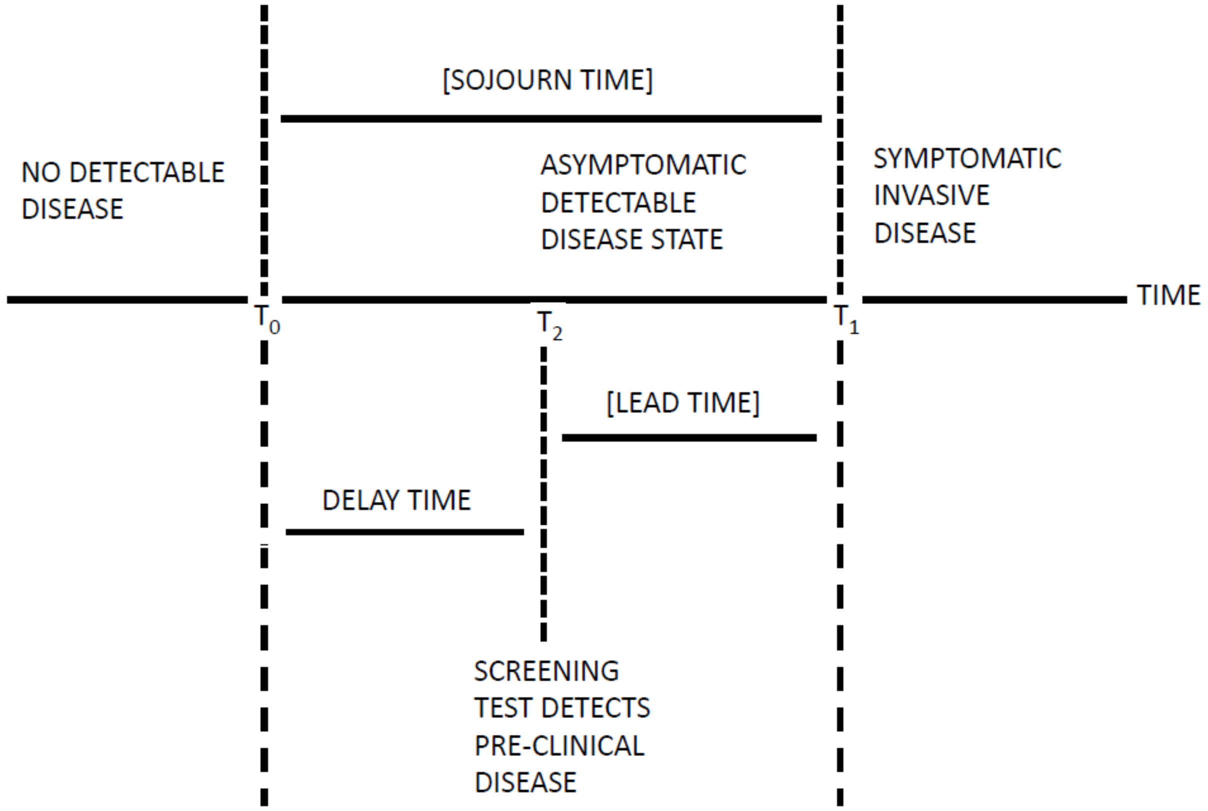

Fig 1. Schema for the progression of a chronic disease, with the intervention of an early detection screening test. [Referred from Reference 10]
